# How Does the Phenol Structure Influence the Results of the Folin-Ciocalteu Assay?

**DOI:** 10.3390/antiox10050811

**Published:** 2021-05-20

**Authors:** Melanie Platzer, Sandra Kiese, Thomas Herfellner, Ute Schweiggert-Weisz, Peter Eisner

**Affiliations:** 1 ZIEL-Institute for Food & Health, TUM School of Life Sciences Weihenstephan, Technical University of Munich, Weihenstephaner Berg 1, 85354 Freising, Germany; peter.eisner@ivv.fraunhofer.de; 2 Fraunhofer Institute for Process Engineering and Packaging IVV, Giggenhauser Str. 35, 85354 Freising, Germany; sandra.kiese@ivv.fraunhofer.de (S.K.); thomas.herfellner@ivv.fraunhofer.de (T.H.); ute.weisz@ivv.fraunhofer.de (U.S.-W.); 3 Chair of Food Science, Institute for Nutritional and Food Sciences, University of Bonn, Meckenheimer Allee 166a, 53113 Bonn, Germany; 4 Faculty of Technology and Engineering, Steinbeis-Hochschule, George-Bähr-Str. 8, 01069 Dresden, Germany

**Keywords:** reducing capacity, antioxidant effect, flavonoids, phenolic acids, structure-activity relationship

## Abstract

Plants produce a diverse array of secondary metabolites that are generally nonessential but facilitate ecological interactions. Fruits, vegetables, seeds and nuts can accumulate bioactive secondary metabolites with health-promoting properties, including the potent antioxidant activities of phenolic compounds. Several in vitro assays have been developed to measure the polyphenol content and antioxidant activity of plant extracts, e.g., the simple and highly popular Folin-Ciocalteu (FC) assay. However, the literature contains a number of different descriptions of the assay and it is unclear whether the assay measures the polyphenol content or reducing capacity of the sample. To determine the influence of phenolic structures on the outcome of the FC assay, we tested phenols representing different subgroups (phenolic acids, flavonols, flavanols, dihydrochalcones and flavanones). We observed different results for each reference substance and subgroup. Accordingly, we concluded that the FC assay does not measure the polyphenol content of a sample but determines its reducing capacity instead. Assigning the substances to five structural classes showed that the FC results depend on the number of fulfilled Bors criteria. If a molecule fulfills none of the Bors criteria, the FC results depend on the number of OH groups. We did not find a correlation with other single electron transfer assays (e.g., ABTS and DPPH assays). Furthermore, the FC assay was compatible with all five subgroups and should be preferred over the DPPH assay, which is specific for extracts rich in dihydrochalcones or flavanones.

## 1. Introduction

Plants produce tens of thousands of secondary (specialized) metabolites that are not required for basic cellular functions or normal growth and development, but fulfill various ecological roles including protection from herbivores, pests, pathogens and UV radiation [1,2,3]. Secondary metabolites that accumulate in the edible parts of plants are known for their health-promoting and disease-preventing properties in humans, attracting interest from the pharmaceutical, food and nutritional supplement industries. Many secondary metabolites are potent antioxidants, with the ability to scavenge reactive oxygen species and other free radicals. These compounds can protect foods from oxidation and are widely used as additives to improve food quality and storage stability [1,4,5,6,7,8].

Phenolic compounds are among the most important antioxidants found in plants. This diverse group includes low-molecular-weight compounds with a single aromatic ring, such as phenolic acids, as well as large and complex polyphenolic molecules, such as flavonoids [9,10]. Two important structural subgroups of the phenolic acids are the hydroxybenzoic acids and the hydroxycinnamic acids, which are found in almost all plant-based foods and are thought to promote protein synthesis and nutrient absorption. The most common phenolic acids are ferulic acid, *p*-coumaric acid, gallic acid and caffeic acid [11,12]. Flavonoids typically consist of two or more aromatic rings, each carrying at least one aromatic hydroxyl group, with the rings connected by a carbon bridge [13]. They are divided into four subgroups (flavonols, flavanols, dihydrochalcones and flavanones) based on the connection between the B- and C-rings as well as the oxidation state and the functional groups on the C-ring [14]. The phenolic compounds considered in this study are summarized in Table 1 (slightly modified from Table 1 in Platzer et al. [15] with permission).

**Figure 1:**
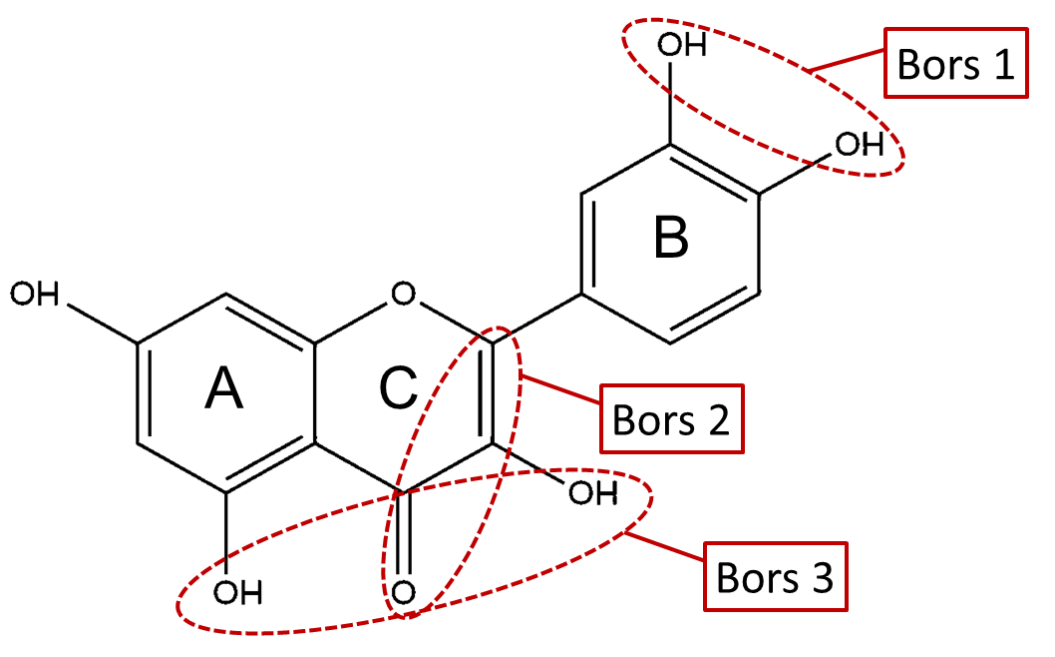
Structure activity relationship based on Bors criteria. Bors 1—catechol group on the B-ring; Bors 2—2,3 double bond and 4-oxo group on the C-ring; Bors 3—OH groups at position 3 and 5 OH group on the A- and C-rings and 4-oxo group on the C-ring.

The antioxidant activity of these compounds depends on their overall structure as well as the position and type of side groups, which is known as the structure-activity relationship (SAR) [16]. Three criteria have been proposed to explain the antioxidant behavior of phenolic compounds, and these are known as Bors criteria [17]. The first is the presence of a catechol group on the B-ring (Bors 1), which increases the stability of the resulting antioxidant radical. The second is the presence of a 2,3 double bond combined with a 4-oxo group on the C-ring (Bors 2), which facilitates electron delocalization. The third is the presence of OH groups at positions 3 and 5 in combination with a 4-oxo group, which enables electron delocalization via hydrogen bonds (Bors 3). The three Bors criteria are summarized in Figure 1.

The antioxidant activity of phenolic compounds is often determined using a rapid assay. Popular tests include the 2,2’-azino-bis (3-ethylbenzothiazoline-6-sulfonic acid) (ABTS), 2,2-diphenyl-1-picrylhydrazyl (DPPH), oxygen radical absorbance capacity (ORAC) and Folin-Ciocalteu (FC) photometric assays [18,19,20,21]. These are based on either single electron transfer (SET) or hydrogen atom transfer (HAT) reaction mechanisms [22,23]. The ABTS, DPPH and FC assays follow a SET mechanism and opinions vary as to whether there is a correlation between the results of these assays [21,23,24,25,26]. Common trends and differences between the ABTS and DPPH assays have been reported previously [15].

The FC assay was originally developed for the detection and quantification of tyrosine [27] and was later modified to measure the polyphenol content of red wine [28].

The assay quickly gained popularity due to its simplicity and reproducibility. When the FC reagent is exposed to an antioxidant, it is reduced to a blue complex that can be quantified by spectrophotometry. The exact composition of the reagent is not published, but it probably consists of a mixture of heteropolyacid, phosphomolybdenum and phosphotungstic acid. The color change is thought to reflect the reduction of molybdenum, as shown in Equations (1) and (2) [23,28].

(1) $$Mo^{+6}\left(yellow\right)+e^{-1}\to Mo^{+5}\left(blue\right)$$

(2) $$Mo^{+5}+e^{-1}\to Mo^{+4}\left(blue\right)$$

In the literature, the FC assay is often described as a means to determine the total polyphenol content and is typically used for the analysis of plant extracts and juices [27,29,30]. The SAR is rarely mentioned because extracts and juices are complex mixtures of many phenolic and nonphenolic components, which makes the effect of particular structures impossible to isolate. Some authors have suggested that FC assay results depend on the number and position of OH groups, particularly those on the B-ring [31,32,33]. The OH group at position 3 on the A-ring also appears to play an important role [31]. Furthermore, OH groups in the *para* or *ortho* positions appear to confer stronger reducing capacity than those in the *meta* position due to the stabilization of the phenoxyl radical by intramolecular hydrogen bonds [16,31,34]. Replacing a hydrogen atom with a methoxy group also increases the reducing capacity, but replacing an OH group with a methoxy group has the opposite effect [16,31,35,36]. Finally, hydroxycinnamic acids produce stronger signals than hydroxybenzoic acids due to the resonance stabilization of the radical, which improves the ability to donate protons [31,37].

To determine the influence of phenolic structures and the total polyphenol content on the outcome of FC assay results in more detail, we selected 24 different standard references representing the phenolic acids, flavonols, flavanols, dihydrochalones and flavanones. We investigated the potential of a new classification system based on reducing capacity. We also compared the results of the FC assay to other SET-based methods to determine which assay correlates most closely with the Bors criteria.

## 2. Materials and Methods

Chemicals and reference standards were obtained from Sigma-Aldrich (Steinheim, Germany): caffeic acid (CAA), (+)-catechin (CAT), 3,4-dihydroxybenzoic acid (DBA), (−)-epicatechin (EPC), ferulic acid (FEA), gallic acid (GAA), 4-hydroxybenzoic acid (HBA), hesperetin (HES), kaempferol (KAE), morin (MOR), naringenin (NAN), p-coumaric acid (PCA), phloridzin (PHD), phloretin (PHT), quercetin-3-D-galactoside (QGA3), quercetin-3-D-glucoside (QGU3), quercetin-7-D-glucoside (QGU7), quercetin (QUR), sinapic acid (SIA), siringic acid (SRA), taxifolin (TAF) and FC reagent. The standard reference narirutin (NAR) was obtained from K&J Scientific (Marbach am Neckar, Germany) and isorhamnetin (IRT) and naringin (NAG) from Carl Roth (Karlsruhe, Germany). Stock solutions were prepared by dissolving the reference standards in analytical grade absolute ethanol and diluting each of them in seven steps for the measurements.

The reducing capacity was determined by spectrophotometry as orignally described [28] with slight modifications. Briefly, 20 μL of each sample (reference standard dilution or ethanol) was mixed with 100 μL demineralized water (H${}_{2}$O${}_{\mathrm{demin}}$) and 100 μL FC reagent and were incubated for 3 min in the dark. We then added 1580 μL of H${}_{2}$O${}_{\mathrm{demin}}$ and 200 μL 7.5% (*w*/*v*) sodium carbonate and incubated for another 30 min in the dark. The absorption of the samples was measured at 765 nm using a Specord 210 plus spectrophotometer (Analytik Jena, Jena, Germany). The slope was determined dy linear regression and presented as the reducing capacity.

For statistical analysis, Sigma Plot (Systat Software, San Jose, CA, USA) was used for one-way analysis of variance (ANOVA) corresponding to an unpaired t-test. If there was a significant difference, an additional pairwise test was carried out using the Holm–Šidák method. The significance level for both tests was 0.05. Statistical analysis was always carried out with all significant decimal places.

## 3. Results and Discussion

### 3.1. Analysis of Individual Reference Standards in the FC Assay

**Figure 2:**
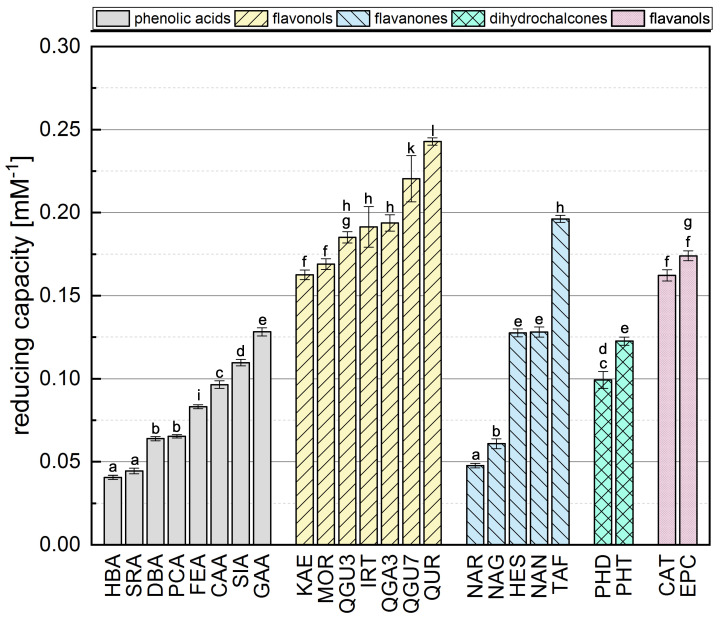
The reducing capacity of all standard reference compounds measured in the FC assay. Equal letters indicate that there is no significant difference between the values. The significance level was 0.05.

Phenolic reference compounds representing five different subgroups, which are shown in Table 1, were tested in the FC assay and the absorption of the reduced complex was quantified by spectrophotometry. The reducing capacities of all the reference compounds (calculated from the absorbance readings) are shown in Figure 2. The ranking was partly in agreement with previous studies [31,38,39,40].

The comparison of phenolic acids showed significantly different results for most of the compounds, with the exception of HBA and SRA as well as DBA and PCA. The presence of a galloyl group had the strongest influence on the reducing capacity, explaining the top-ranking position of GAA. We also found that a hydroxycinnamic acid group is more important than a hydroxybenzoic acid group, probably because the former enhances resonance stabilization [31,35,41]. Accordingly, SIA achieved a higher reducing capacity than SRA, PCA a higher value than HBA, and CAA a higher value than DBA. Furthermore, a higher reducing capacity was observed for compounds with a catechol group instead of an OH group at position 4, explaining why CAA showed a higher reducing capacity than PCA and FEA, and why DBA and GAA achieved higher values than HBA. Finally, the reducing capacity was also increased by the presence of an additional methoxy group, which is why SRA achieved a slightly higher value than HBA, and SIA a higher value than FEA and PCA. A methoxy group at position 5 appeared to confer greater reducing capacity than one at position 3, explaining the higher reducing capacity of DBA compared to SRA, and FEA compared to CAA. Replacing a hydrogen atom with a methoxy group, which is an electron donor, can reduce the bond dissociation energy, promote electron transfer, and increase the reducing capacity [16,31,35,36].

Among the flavonols, QUR achieved the highest reducing capacity because it fulfills all three Bors criteria. QGU7 was significantly less active because the OH at position 7 is replaced by a glucoside. In contrast to phenolic acids, the catechol group on the B-ring (corresponding to Bors 1) had the strongest influence on the reducing capacity of flavonols, explaining the high values observed for QUR, QGU7, QGA3 and QGU3. Furthermore, reducing capacity was increased by the presence of an OH group rather than a sugar at position 3, explaining the higher value observed for QUR compared to QGU3 and QGA3. Replacing a hydrogen atom with an electron donating group increases the reducing capacity, but replacing an OH group with an less electron donating group, such as a sugar, has the opposite effect [42]. The presence of an OH group at position 4’ had a significant effect whereas an OH group at position 2’ had a negligible effect, which is why there was no significant difference between MOR and KAE. An additional methoxy group again enhanced the reducing capacity, which is why IRT achieved a higher value than KAE. QGU3 and QGA3 were not significantly different and we assume that the type of sugar residue does not influence the reducing capacity.

The presence of a catechol group (Bors 1) also appeared to exert the strongest influence on the reducing capacity of flavanones, explaining why TAF achieved a higher value than HES, NAR and NAN. TAF also fulfills Bors 3, which contributes to its top-ranking position (in contrast to the other substances), so more research is needed to determine the influence of this structure. Although HES has an additional methoxy group on the B-ring, its reducing capacity was not significantly different to that of NAN. An OH group in the *para* position therefore appears to exert a greater influence than one in the *meta* position with an additional methoxy group. The presence of an OH group at position 7 also had a strong influence, which is why the reducing capacity of NAN was significantly higher than for NAR. As stated above for the flavonols, the type of sugar residue appeared not to influence the reducing capacity, explaining the similar values observed for NAR and NAG.

In the dihydrochalcone subgroup, PHT achieved a significantly higher value than PHD reflecting the additional OH group at position 4’. No general conclusions could be drawn because we only tested two different compounds. Similarly we only tested two flavanols (structural isomers) and the reducing capacity was similar for both compounds.

### 3.2. Consolidated Analysis of Phenolic Subgroups in the FC Assay

**Figure 3:**
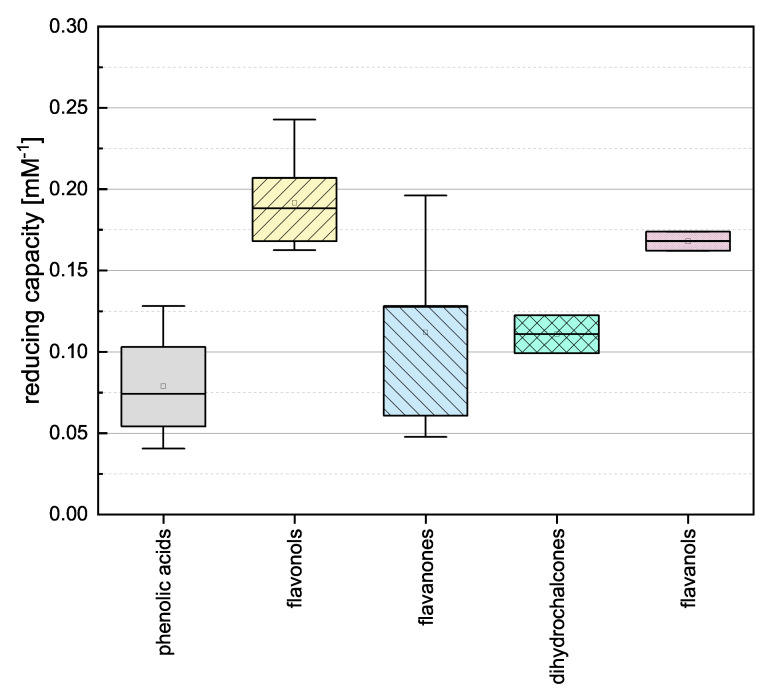
Boxplot of the mean reducing capacities of different subgroups of phenolic compounds (phenolic acids, flavonoles, flavanones, dihydrochalcones, flavanoles) in the FC assay. Error bars represent range within standard errors (1.5 interquartile range).

To gain insight into the general properties of the phenolic subgroups, we combined the individual assay results and evaluated the groups by mean reducing capacity as shown in Figure 3.

The phenolic acids, flavanones and dihydrochalcones showed the lowest mean values and there was no significant difference between them. This is probably because the compounds in these three categories feature the lowest number of OH groups (phenolic acids 1–3, flavanones 2–5, and dihydrochalcones 3–4) and most of them do not meet any of the Bors criteria (the only exception is TAF). The flavanols and flavonols showed higher reducing capacities and there was no significant difference between them. This was anticipated because they feature the highest number of OH groups (flavanols 5 and flavonols 4–5), all flavonols fulfill Bors 3, and some also fulfil Bors 1 and 2. Because the flavanols only fulfil Bors 1 yet are equivalent in value to the flavonols, we assume Bors 1 has the greatest influence on reducing capacity.

### 3.3. Reclassification of the Flavonoid Reference Standards Based on Their Structural Features

**Figure 4:**
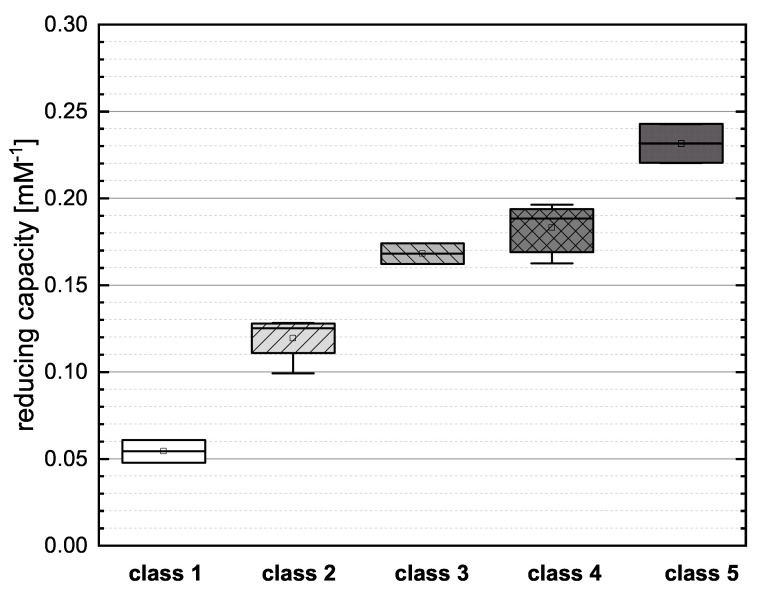
Boxplot of the mean values of the reducing capacity for the new classification of flavonoids as shown in Table 2, investigated in the FC assay. Error bars represent range within standard errors (1.5 interquartile range).

To evaluate the influence of particular structural features in more detail, we assigned the flavonoids reference standards to an alternative classification based on the number of OH groups and the satisfaction of Bors criteria (Table 2). The phenolic acids do not meet the Bors criteria because these were established only for flavonoids and therefore, we have not included them in our reclassification. We then combined the individual assay results and evaluated the new categories by mean reducing capacity as shown in Figure 4.

Class 1 (NAR and NAG) showed the lowest mean value in the boxplot, reflecting the low number of OH groups and the failure to meet any of the Bors criteria. Both substances presented similar values for reducing capacity, suggesting that the type of sugar at position 7 does not have a major influence. In class 2 (PHD, PHT, HES and NAN), PHD and NAN possess three OH groups but do not meet any Bors criteria and their reducing capacities were therefore low. The open-chain structure of PHD and PHT also appears to limit their reducing capacity. PHT and HES achieved similar values, so the position of the OH group appears to play only a minor role. We observed no significant difference between the two representatives of class 3 (the structural isomers CAT and EPC), indicating that the spatial arrangement of the molecule does not affect its reducing capacity. There were no significant differences between the substances in class 4 (KAE, MOR, QGU3, QGA3, IRT and TAF). All of the compounds fulfilled two of the three Bors criteria but not always the same two, so the effect of fulfilling the Bors criteria seems to be nonspecific and additive in this case. Furthermore, there was no significant difference between substances with different numbers of OH groups. Finally, class 5 (QGU7 and QUR) showed the highest mean value in the boxplot because both reference compounds fulfilled all three Bors criteria and also featured the highest number of OH groups.

### 3.4. Comparison of the FC Assay with the DPPH and ABTS Assays

**Figure 5:**
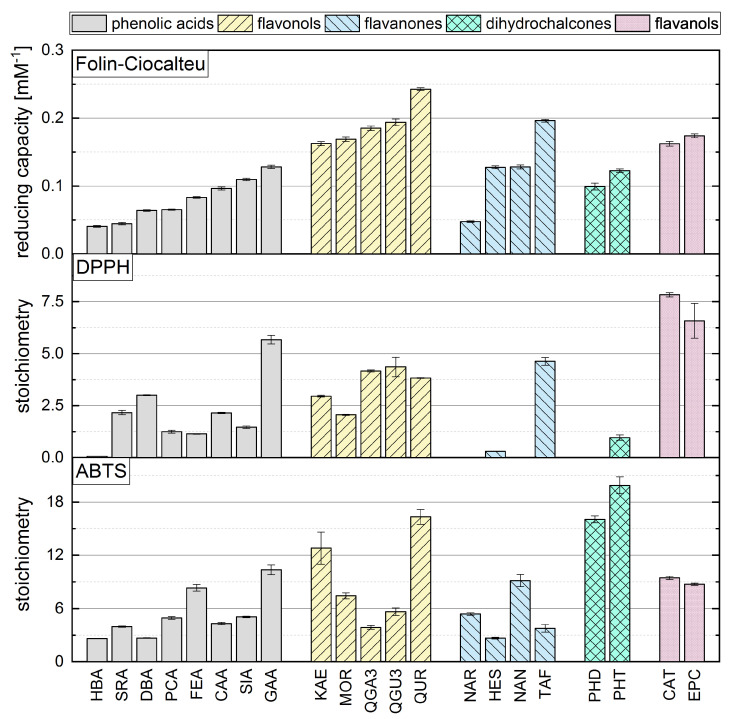
The reducing capacity of all standard references compounds measured in the FC assay in comparison to DPPH and ABTS assays, which are reproduced with permission from Platzer et al. (2021) [15].

Although some authors have reported a good correlation between the results of different in vitro antioxidant assays, others have reported significant discrepancies [21,23,24,25,26,43]. We therefore compared our FC results to our previously reported ABTS and DPPH assay data (Figure 5), where we used the same concentrations, temperature and measurement duration [15]. We observed no clear correlation between the assays, and the antioxidant activity seemed to depend on different criteria in each case. In contrast to the ABTS assay, the FC and DPPH assays appeared to depend mainly on the number of OH groups and the Bors criteria, with Bors 1 showing the greatest influence. The highest reducing capacity was observed for compounds meeting all three Bors criteria, which was not the case in the ABTS assay. However, an OH group in the *para* position of the B-ring appeared to play a greater role than the *meta* or *ortho* position in all three assays.

Among the phenolic acids, the hydroxycinnamic acids achieved higher values than the hydroxybenzoic acids in the FC and ABTS assays, but not in the DPPH assay. Furthermore, substances with an additional methoxy group achieved higher values in all three assays. In the ABTS assay, reference compounds with OH and methoxy groups achieved even higher values than compounds with a catechol group, which was not the case in the FC and the DPPH assays. However, the outcome of the DPPH and FC assays depended mainly on the number of OH groups, whereas the presence of a galloyl group determined the result of the ABTS assay regardless of whether one or two OH groups was present. The DPPH and FC assay results for flavonols depended mainly on the Bors criteria. Substances in this subgroup always satisfy Bors 2, so the influence of Bors 1 and 3 should be considered. Bors 1 had the greatest influence in the DPPH and FC assays but not the ABTS assay. If a galloyl group replaces a catechol group on the B-ring, this only increased the value in the DPPH assay. In contrast, Bors 3 seemed to influence all three assays, because substances with a sugar residue at position 3 achieved lower values but the type of sugar was not important. Furthermore, an additional OH group in *ortho* position on the B-ring did not affect the result of any of the assays.

Bors 1 and 2 play a key role in all three assays when applied to flavanones. Furthermore, an OH group in the *para* position of the B-ring also increases the reducing capacity. The presence of a sugar residue had no influence on the FC assay and slightly worsened the value reported in the ABTS assay but inhibited the reaction in the DPPH assay.

For the dihydrochalcones, PHT achieved a higher value than PHD in all assays, reflecting the presence of an additional OH group. PHD did not react with the radical in the DPPH assay. There was no significant difference between the structurally isomeric flavanols CAT and EPC in any of the assays, indicating that the three-dimensional arrangement of these molecules does not influence the outcome.

## 4. Conclusions

The standard reference compounds measured in the FC assay produced significantly different values, indicating (as previously suggested) that the assay measures reducing capacity rather than the total polyphenol content. The ranking of reference compounds in the FC assay was partly in agreement with the literature and suggested that the reported reducing capacity depends on the SAR, as determined by the number and position of OH groups as well as the satisfaction of Bors criteria. By assigning the reference compounds to five newly-defined classes, we found that the results of the FC assay depend primarily on how many of the Bors criteria are met, with the number of OH groups becoming relevant only if the molecule fails to fulfill any of the Bors criteria. Correlation with other SET-based assays (ABTS and DPPH) is often reported in literature, but we found no clear relationship between them. Interestingly, the three assays appeared to depend (at least in part) on the same structural features, but with different outcomes. One feature common to all three assays was the greater influence of an OH group in the *para* position rather than the *meta* position. The FC assay also appeared to correlate better with the Bors criteria, and thus the SAR of the antioxidants, compared to the other two assays. Accordingly, the antioxidant effect of phenolic compounds in the FC assay can be predicted based on their structural characteristics. Furthermore, all the reference compounds could be detected in the FC assay, whereas the DPPH assay did not show a reaction for some flavanones and dihydrochalcones. Therefore, the FC assay should be preferred especially for extracts rich in these substances. In order to compare the results of the FC assay with the antioxidant activity in specific applications, a correlation in the final medium (e.g., plant oil or food matrix) should be clarified. In addition, other relevant parameters such as pH, solvent, the content of other components (e.g., sugars and proteins) and synergistic effects between different phenolic compounds should be considered when extracts are measured, as these may influence the reported activity. These aspects require more detailed analysis in future experiments.

## Figures and Tables

**Table 1:** The phenolic compounds analyzed in this study (phenolic acids and subgroups of flavonoids) along with reference standards, character codes, and side groups (reproduced and slightly modified from Table 1 in Platzer et al. [15] with permission).

Subgroup	Reference Standard	Sample Code	Class	Side Group						
**phenolic acids**				**1**	**3**	**4**	**5**			
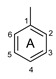	caffeic acid	CAA	-	(CH${}_{2}$)${}_{2}$COOH	OH	OH	H			
	3,4-dihydroxybenzoic acid	DBA	-	COOH	OH	OH	H			
	ferulic acid	FEA	-	(CH${}_{2}$)${}_{2}$COOH	OH	OCH${}_{3}$	H			
	gallic acid	GAA	-	COOH	OH	OH	OH			
	4-hydroxybencoic acid	HBA	-	COOH	H	OH	H			
	*p*-coumaric acid	PCA	-	(CH${}_{2}$)${}_{2}$COOH	H	OH	H			
	sinapic acid	SIA	-	(CH${}_{2}$)${}_{2}$COOH	OCH${}_{3}$	OH	OCH${}_{3}$			
	siringic acid	SRA	-	COOH	OCH${}_{3}$	OH	OCH${}_{3}$			
**flavonols**				**2’**	**3’**	**4’**	**5’**	**3**	**5**	**7**
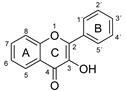	isorhamnetin	IRT	4	H	OH	OH	H	OH	OH	OH
	kaempferol	KAE	4	H	H	OH	H	OH	OH	OH
	morin	MOR	4	OH	H	OH	H	OH	OH	OH
	quercetin-3-D-galactoside	QGA3	4	H	OH	OH	H	Glc	OH	OH
	quercetin-3-D-glucoside	QGU3	4	H	OH	OH	H	Gal	OH	OH
	quercetin-7-D-glucoside	QGU7	5	H	OH	OH	H	OH	OH	Glc
	quercetin	QUR	5	H	OH	OH	H	OH	OH	OH
**flavanones**				**3’**	**4’**	**3**	**5**	**7**		
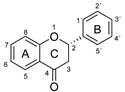	hesperetin	HES	2	OH	OCH${}_{3}$	H	OH	OH	
	narirutin	NAR	1	H	OH	H	OH	2 Glc		
	naringin	NAG	1	H	OH	H	OH	Rham, Glc		
	naringenin	NAN	2	H	OH	H	OH	OH		
	taxifolin	TAF	4	OH	OH	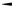 OH	OH	OH		
**dihydrochalcones**				**5**	**7**	**9**	**4’**			
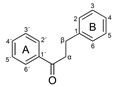	phloridzin	PHD	2	OH	OH	OH	Glc			
	phloretin	PHT	2	OH	OH	OH	OH			
**flavanols**				**3’**	**4’**	**3**	**4**	**5**	**7**	
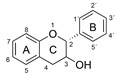	(+)-catechin	CAT	3	OH	OH	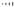 OH	H	OH	OH	
	(-)-epicatechin	EPC	3	OH	OH	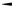 OH	H	OH	OH	

**Table 2:** Reclassification of flavonoids based on their structural features.

Class	Structural Feature	Substances
1	2 OH groups and none of the Bors criteria	NAG, NAR
2	3 to 4 OH groups and none of the Bors criteria	HES, NAN, PHD, PHT
3	one of the Bors criteria	CAT, EPC
4	two of the Bors criteria	IRT, KAE, MOR, QGA3, QGU3, TAF
5	three Bors criteria	QUR, QGU7

## Data Availability

Data of the measurement results are available from the authors.

## Abbreviations

The following abbreviations are used in this manuscript:

**ABTS**	2,2’-azino-bis (3-ethylbenzothiazoline-6-sulfonic acid)
**CAA**	caffeic acid
**CAT**	(+)-catechin
**DBA**	3,4-dihydroxybenzoic acid
**DPPH**	2,2-diphenyl-1-picrylhydrazyl
**EPC**	(−)-epicatechin
**FC**	Folin-Ciocalteu
**FEA**	ferulic acid
**GAA**	gallic acid
**HAT**	hydrogen atom transfer
**HBA**	4-hydroxybenzoic acid
**HES**	hesperetin
**IRT**	isorhamnetin
**KAE**	kaempferol
**MOR**	morin
**MYR**	myricetin
**NAG**	naringin
**NAN**	naringenin
**NAR**	narirutin
**ORAC**	oxygen radical absorbance capacity
**PCA**	*p*-coumaric acid
**PHD**	phloridzin
**PHT**	phloretin
**QGA3**	quercetin-3-D-galactosides
**QGU3**	quercetin-3-D-glucosides
**QGU7**	quercetin-7-glucoside
**QUR**	quercetin
**SIA**	sinapic acid
**SAR**	structure-activity relationship
**SET**	single electron transfer
**SRA**	siringic acid
**TAF**	taxifolin
